# Comprehensive analysis of physiological characteristic and global transcriptional profiling to unravel the mechanism of wheat yield increase in intercropping

**DOI:** 10.3389/fpls.2026.1857726

**Published:** 2026-08-10

**Authors:** Yang Chen, Hong Fan, Cai Zhao, Xiaoyuan Bao, Congcong Guo, Yali Sun, Bo Jing, Wei He

**Affiliations:** State Key Laboratory of Aridland Crop Science, Gansu Agricultural University, Lanzhou, China

**Keywords:** intercropping, molecular mechanism, physiological traits, transcriptome, wheat

## Abstract

**Introduction:**

Wheat (*Triticum aestivum* L.) is a major staple crop for billions of people. Intercropping can increase wheat grain yield and contribute to food security. However, the physiological characteristics and molecular basis underlying yield increases in intercropped wheat remain insufficiently understood.

**Methods:**

This study integrated physiological measurements with global transcriptional profiling of wheat leaves and roots to investigate responses associated with wheat yield increase under intercropping relative to sole cropping.

**Results:**

Intercropping increased wheat grain yield by 19.12% and 14.94% and spike number per unit area by 27.4% and 25.3% in 2020 and 2021, respectively, whereas grain number per spike and thousand-grain weight were not significantly affected. Intercropping also increased relative chlorophyll content (SPAD value), net photosynthetic rate (Pn), leaf soluble protein content, peroxidase (POD) activity, and sucrose-phosphate synthase (SPS) activity, while decreasing malondialdehyde (MDA) content in wheat leaves. RNA sequencing (RNA-seq) analysis identified 1993 differentially expressed genes (DEGs) in leaves, including 1005 upregulated and 988 downregulated genes, and 17866 DEGs in roots, including 10782 upregulated and 7084 downregulated genes. Upregulated DEGs were mainly enriched in protein processing in the endoplasmic reticulum, plant–pathogen interaction, and photosynthesis–antenna proteins in leaves, and in phenylpropanoid biosynthesis, glutathione metabolism, and alpha-linolenic acid metabolism in roots. The expression trends of 15 of the 16 selected DEGs were consistent between RNA-seq and quantitative reverse-transcription PCR (qRT-PCR) analyses.

**Discussion:**

Taken together, the increased biomass accumulation and grain yield of intercropped wheat may be associated with coordinated changes in secondary-metabolite-related pathways, plant–pathogen interactions, protein synthesis and processing, and photosynthesis-related processes. These findings advance our understanding of the physiological and transcriptional responses of wheat to intercropping, identify candidate processes and pathways associated with its yield advantage, and provide a basis for further functional validation and breeding research in intercropping systems.

## 1 Introduction

Intercropping is the deliberate blending of various crop types in a single field (Willey, 1990), which has been applied worldwide because of high productivity and yield stability (Raseduzzaman and Jensen, 2017; Li et al., 2020a). Also, intercropping has significant ecological functions, such as increasing resource use efficiency and augmenting soil carbon and nitrogen content (Willey, 1990; Hu et al., 2017a). It can improve agricultural resiliency and environmental sustainability at the same time.

To maximize the benefits of intercropping, it’s often combining species with different functional traits, e.g., a C3 with a C4 species, because the photosynthesis performance, water and nitrogen use efficiency, and growth period in C3 and C4 species are different (Sage et al., 1987; Taylor et al., 2011). These features that distinguish C3 from C4 species may work in concert to optimize system function. For example, cereals/legumes intercropping is strongly suggested in low-input agriculture because it can improve nitrogen-use efficiency (Mousavi and Eskandari, 2011; Pelzer et al., 2014). However, legume crops in the intercropping system often performed as yield loss. For instance, mung bean (Vigna radiata L.) intercropped with proso millet (Panicum miliaceum L.) showed a significant decrease in grain yield per plant compared with sole cropping (Gong et al., 2020), and peanut (Arachis hypogaea L.)/maize (Zea mays L.) intercropping decreased the partial land equivalent ratio (LER) of peanut (Feng et al., 2021). Whereas appropriate cereals/cereals intercropping can achieve overyielding of both intercropped crop components and increase farmers’ income (Chai et al., 2021). The biomass yield advantage in maize/legume intercropping in absolute terms (grain yield per unit area) is up to 2.43 t·ha^-1^, according to a meta-analysis on China national scale (Li et al., 2020a). Wheat is one of the globally most important crops as a staple food for billions of people and its high yield is globally essential for food security (Voss-Fels et al., 2019). Wheat-maize intercropping is a traditional cropping pattern in northwest China, where is rich in radiation resources and suitable for developing intercropping (Yin et al., 2020). Wheat and maize are C3 and C4 crops, respectively, and differ in their growth periods and requirements for light and temperature. Border wheat rows contributed substantially to this yield advantage because they received more favorable light, nutrient, and moisture conditions than inner rows (Fan et al., 2023). These trait differences between wheat and maize may synergize to maximize shoot and root functioning (Wang et al., 2018; Gong et al., 2020). Besides, their variances in tolerance to climate conditions maximize their growth and yield performance (Yin et al., 2021). This intercropping system produced a grain yield of 13.84 t ha^–1^, and wheat grain yields were increased by 36.5% compared with sole-cropped wheat (Chai et al., 2021).

This outstanding yield performance of intercropped wheat is related to above-ground and below-ground interspecific interactions between crop species (Wang et al., 2018; Yin et al., 2021). First, wheat was sown earlier than maize so it gained a competitive advantage in radiation capture in the intercropping system, and wheat’s radiation-use efficiency (RUE) and leaf-area development were higher than those under sole cropping (Gao et al., 2014; Gou et al., 2017; Wang et al., 2017b). Moreover, nutrient and water acquisition by intercropped wheat increased significantly than sole-cropped wheat (Li et al., 2001; Fan et al., 2013; Gao et al., 2014; Hu et al., 2017a). These interspecific interactions between crop species also contribute to wheat’s physiological traits and growth. Intercropped wheat has an increased dry matter accumulation (Gao et al., 2014; Yin et al., 2020) and number of tillers per plant and kernels per ear (Gou et al., 2016). Wang et al. (2018) indicated that wheat roots were more developed in intercropping than sole cropping. Wheat’s photosynthetic capacity and leaf water potential were optimized by intercropping (Li et al., 2020b; Yin et al., 2021). Meanwhile, wheat growth and yield formation are influenced by cellular enzymes and metabolites in leaves, which have been less frequently studied. For example, sucrose-phosphate synthase (SPS) regulates the balance between sucrose and starch and influences the rate of sucrose export from source tissues (Wang et al., 2017a), whereas peroxidase (POD) activity and malondialdehyde (MDA) content reflect the capacity of the antioxidant system to resist oxidative damage (Wang et al., 2015). Furthermore, a series of downstream physiological and metabolic processes are activated by environment-responsive genes through a complicated signal transduction network (You and Chan, 2015; Alves et al., 2022). A comparative transcriptomic analysis of maize rhizospheres under monocropping and intercropping identified distinct responses involving stress signaling, secondary metabolism, and carbon–nitrogen regulation (Zhang et al., 2024). However, the tissue-specific transcriptional responses of wheat leaves and roots to wheat–maize intercropping remain insufficiently understood.

The objectives of this study were to (1) evaluate the yield performance of intercropped wheat under irrigated conditions, (2) investigate the physiological and biochemical characteristics of wheat, and (3) reveal the molecular mechanism of high yield of intercropped wheat. Information on these subjects is essential for a better understanding of the intercropping system, which will help management decisions.

## 2 Materials and methods

### 2.1 Test area description

The field experiment was conducted in 2020 and 2021 at the Oasis Agricultural Experiment Station of Gansu Agricultural University (37.96°N, 102.64°E) in Wuwei City, Gansu Province, China. The station is located in the eastern side of the Hexi Corridor, a temperate arid region with a continental climate. Mean annual precipitation in the past 20 years has been less than 180 mm. Over the same period, mean annual air temperature was below 7.2 °C, and accumulated temperature above 10°C was approximately 2,900°C, meeting the thermal requirements of wheat-maize intercropping. The experimental site is representative of an irrigated agroecosystem in an arid region.

### 2.2 Experimental design

The treatments consisted of wheat–maize relay intercropping (IW) and sole-cropped wheat (SW), with SW serving as the control. In 2020-2021, both crops were planted at each experimental year with sowing and harvesting dates consistent with all treatments; wheat was sown on 21 and 24 March and harvested on 22 and 23 July; maize was sown on 19 and 20 April and harvested on 28 and 27 September. Each treatment had three replicates. Each plot was 51.3 m^2^ (9.0 m×5.7 m) with a 0.5 m wide by 0.3 m high ridge between two neighboring plots to eliminate potential movement of water and nutrition.

Field wheat (cv. Ning-chun 4) and maize (cv. Xian-yu 335) were applied in this experiment. In the wheat-maize intercropping plots, wheat and maize were alternated in 1.9 m-wide strips, and three wheat-maize intercropping strips constituted one intercropped plot. In each intercropped plot, 8/19 was occupied by wheat, and the remaining 11/19 was occupied by maize. Each intercropped plot consisted of three pairs of wheat and maize strips, and each wheat strip consisted of six rows of wheat, and each maize strip consisted of three rows of maize. The row spacing of wheat and maize in the intercropping system was 0.12 m and 0.4 m, respectively.

In sole cropping and intercropping systems, wheat and maize’s the nitrogen and phosphorus application ratios were consistent under the same land area. The phosphorus amount (P_2_O_5_) was 90 kg·ha^-1^ and 180 kg·ha^-1^ for wheat and maize, respectively. The nitrogen amount (N) was 180 kg·ha^-1^ and 360 kg·ha^-1^ for wheat and maize, respectively. The irrigation amount was 2400 m^3^·ha^-1^.

### 2.3 Data collection

#### 2.3.1 Aboveground dry matter, grain yield and harvest index of wheat

In 2020-2021, at wheat physiological maturity, the aboveground dry matter was measured from 20 wheat plants that were randomly sampled and cut at the soil surface to reduce sampling error. All biomass samples were oven-dried at 105°C for 1 h for desiccation and then at 80°C until they reached constant mass. Dry mass was measured using an electronic balance.

All wheat plants within a 2.0 m² harvest area in each plot were harvested and threshed separately. The moisture content of the harvested grain was measured immediately after threshing using a portable grain moisture meter (PM-8188-A, KETT Co., Ltd., Japan). Grain yield was adjusted to a standard moisture content of 14% and converted to kg ha^-1^ using the following equation:


$$Y=W\times \left(100-M\right)/86\times 5000$$

where *Y* is the grain yield at 14% moisture content (kg·ha^-1^), *W* is the harvested grain weight from the 2.0 m² sampling area (kg), and *M* is the measured grain moisture content (%). The harvest index (HI) was calculated by the following equation (Donald and Hamblin, 1976).

HI = Grain yield/Aboveground dry matter (DM).

Yield components were also determined at physiological maturity. Productive spikes were counted in two adjacent 1-m row sections in each plot, and the results were expressed as spike number per unit area. Twenty representative spikes were randomly collected from each plot to determine the grain number per spike. Thousand-grain weight was determined by weighing 1000 randomly selected grains using an electronic balance. All measurements were conducted using three independent field replicates.

#### 2.3.2 Chlorophyll relative content (SPAD) and photosynthetic rate (Pn) of wheat leaves

The relative chlorophyll content of wheat leaves was detected with the handy plant chlorophyll meter (SPAD-502 Plus Chlorophyll Meter, Konica Minolta, Japan) at the wheat jointing, booting, and grain filling stage in 2020-2021. Top fully expanded leaves at the wheat jointing stage, and flag leaves at the booting and grain filling stage were selected to be measured. The SPAD value was determined at the middle of each selected leaf, and the mean value of three measurements was calculated to represent the SPAD value of each plot.

The net photosynthetic rate (Pn) was measured with a portable photosynthesis system (LI-6800XT; LI-COR, Lincoln, NE, United States). Measurements were taken on a sunny morning (9:00-11:00 a.m.) to avoid potential stomatal closure around noontime. The measurement time, sampling dates, leaf positions, and selected leaves were consistent with those used for SPAD measurements.

#### 2.3.3 Wheat physiological and biochemical traits

##### 2.3.3.1 Wheat leaves and roots sampling

In 2021, the leaf samples were collected between 9:00-11:00 a.m. on a sunny day from top fully expanded leaves at the wheat jointing stage, and from wheat flag leaves at booting and grain filling stage, collecting one leaf without disease and pest from each plant from 20 plants per treatment. Leaves were cut from the base, without leaf sheath. The collected leaves were rinsed with distilled water and then gently dried with absorbent paper. Aluminum foil was used for packaging the clean leaves, which were then put in an ultralow temperature freezer at -80°C after being frozen in liquid nitrogen. The following parameters were subsequently measured whenever possible. The sample of leaves and roots from 20 plants per treatment were collected at the wheat booting stage for transcriptomic analysis and quantitative RT-PCR validation. LIW and RIW denote leaves and roots, respectively, of wheat grown under intercropping, whereas LSW and RSW denote leaves and roots, respectively, of sole-cropped wheat.

##### 2.3.3.2 Determination of physiological and biochemical indices

###### 2.3.3.2.1 Malondialdehyde content

MDA, a biomarker for oxidative stress, was extracted and quantified spectrophotometrically using a thiobarbituric acid (TBA) procedure (Bao et al., 2009). First, a 200 mg leaf sample was pulverized and extracted in 5 mL trichloroacetic acid (TCA) and rotated for 10 min at 4°C. Then it was heated in boiling water with 0.6% TBA for 15 min and centrifuged at 12000 rpm for 10 min at 4°C. Absorbance was measured at 450, 532, and 600 nm using a UV–visible spectrophotometer.

###### 2.3.3.2.2 Leaf soluble protein content

The amount of leaf soluble protein was determined using the Bradford method (Bradford, 1976). This approach relies on the hypochromic effect from 465 to 595 nm caused by the dye Coomassie Brilliant Blue G-250 specific binding to proteins containing basic and aromatic amino acids. Using a standard curve, the total protein content was calculated from bovine serum albumin and expressed as mg·g^-1^ fresh weight.

###### 2.3.3.2.3 Peroxidase activity

The Kar and Mishra protocol (Kar and Mishra, 1976) was used to measure the POD activity. The reaction mixture contained 5 mL of 10 mM pyrogallol, 5 mL of 5 mM H_2_O_2_, 100 μL of enzyme extract, and 5 mL of 0.1 mM Tris-HCl buffer. POD activity was measured by the decrease in absorbance caused by the H_2_O_2_-dependent oxidation of pyrogallol at 425 nm, which was represented as POD IU·minute^-1^·g^-1^ fresh weight.

###### 2.3.3.2.4 Sucrose phosphate synthase activity

Frozen samples (500 mg) were ground in an extraction solution (5 mL) to measure SPS activity. This extraction solution contained the following ingredients: 10 mM MgCl_2_, 1 mM phenylmethylsulfonyl fluoride, 2% polyvinyl pyrrolidone, 1 mM ethylene glycol bis-(2-aminoethyl ether)-tetraacetic acid, 1% (v/v) Triton X-100, 1 mM ethylenediaminetetraacetic acid, 0.5% (w/v) bovine serum albumin, and 50 mM N-(2-hydroxyethyl) piperazine-N’-(2-ethane sulfonic acid)-NaOH buffer (pH 7.5). The supernatant was collected after centrifuging the homogenate for 15 min at 15000 g at 4 °C. According to Wang et al (Wang et al., 2017a), sucrose synthesis was evaluated to determine the SPS activity, which was represented as SPS IU·minute^-1^·g^-1^ fresh weight.

#### 2.3.4 Transcriptomic analysis of wheat leaves and roots

Flag leaves and roots of wheat at the booting stage in 2021 were collected for transcriptome sequencing. Total RNA of leaves and roots was extracted using an RNAprep Pure Plant Kit (Tiangen, China). On an Agilent 2100 Bioanalyzer (Agilent Technologies, CA, United States), the extracted RNA’s integrity was examined. Origin-gene Biotech Co., Ltd. (Shanghai, China) constructed the cDNA library and performed the transcriptome sequencing on an Illumina HiSeq 2500 platform, producing paired-end reads. Raw reads were filtered using Trimmomatic v0.36 to remove adapter sequences and low-quality reads. The clean reads were aligned to the *Triticum aestivum* IWGSC reference genome from Ensembl Plants release 45 using HISAT2 v2.2.1.0. Gene-level read counts were obtained using HTSeq-count v0.9.1, and gene expression levels were expressed as fragments per kilobase of transcript per million mapped reads (FPKM). Differential expression between LIW and LSW and between RIW and RSW was analyzed using DESeq2 v1.20.0 based on normalized read counts and a negative binomial model. The resulting *P*-values were adjusted using false discovery rate correction, and genes with |log2 fold change| > 1 and a q-value < 0.05 were considered differentially expressed genes (DEGs). The functions of the identified genes were annotated against public databases, including the NCBI non-redundant protein database (NR), Swiss-Prot, the Kyoto Encyclopedia of Genes and Genomes (KEGG), the euKaryotic Orthologous Groups database (KOG), and Gene Ontology (GO), using an E-value threshold of 1×10^-5^. GO terms were assigned based on the Swiss-Prot annotations for GO classification, and putative metabolic pathways were annotated using the KEGG database.

#### 2.3.5 Quantitative RT-PCR validation

A total of 16 representative upregulated DEGs associated with significantly enriched pathways in wheat leaves and roots were selected for qRT-PCR validation. The PrimeScript™ RT reagent Kit with gDNA Eraser (Perfect Real Time) (TaKaRa, Dalian, China) was used to synthesize cDNA from total RNA following the manufacturer’s recommendations. The qRT-PCR was performed according to Duan (Duan et al., 2020) and GAPDH (CuGenDB number:MELO3C008219) was used as reference gene. Each sample underwent qRT-PCR analysis in triplicate. Primer Premier 5.0 software was used to design the specific primers. All primers used in this study are listed in the **Supplementary Table 1**.

### 2.4 Statistical analysis

All physiological, biochemical, agronomic, and yield-component data are presented as means ± standard deviations of three independent field replicates. Differences between intercropped and sole-cropped wheat were analyzed separately for each year and growth stage using one-way analysis of variance (ANOVA) in SPSS 22.0 (IBM Corp., Armonk, NY, United States). Duncan’s multiple range test was used for mean comparisons, and differences were considered significant at *p* < 0.05.

## 3 Results

### 3.1 Effects of cropping patterns on physiological and biochemical, growth characteristics and yield of wheat

**Figure 1:**
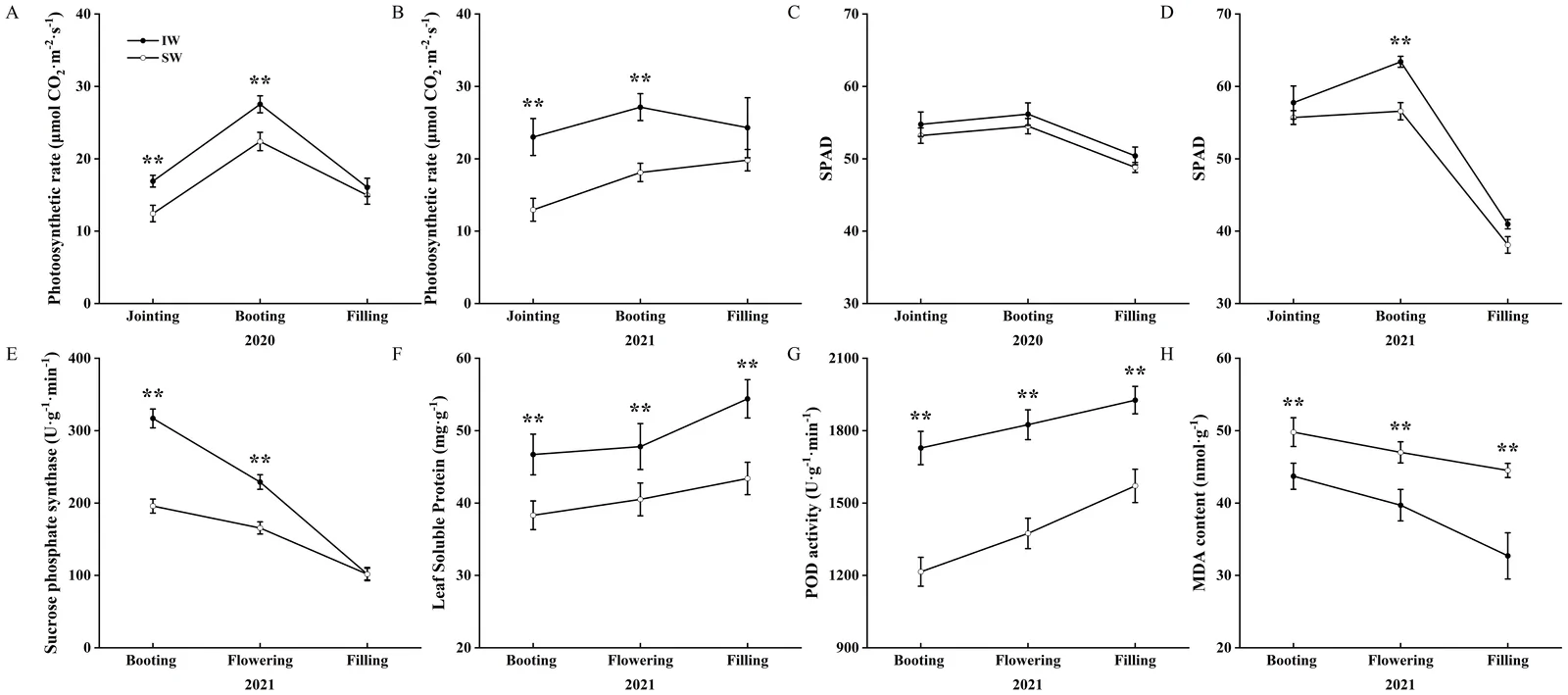
The physiochemical changes in leaves of wheat under different cropping patterns. **(A, B)**, Photosynthetic rate in 2020 and 2021; **(C, D)**, SPAD in 2020 and 2021; **(E)**, Sucrose phosphate synthase activity; **(F)**, Leaf soluble protein content; **(G)**, POD activity; **(H)**, MDA content. The values are means ± SD from three biological replicates (**, *p*<0.01).

The 2-year experiment indicated that the photosynthetic rate and SPAD in intercropped and sole-cropped wheat increased first and then decreased from the jointing to grain filling stage, whereas the photosynthetic rate and SPAD in the leaves of intercropped wheat maintained a higher level than those of sole-cropped wheat (**Figures 1A–D**). Furthermore, the cropping patterns significantly changed in soluble protein and sucrose phosphate synthase activity in wheat leaves. However, the contents of soluble protein and sucrose phosphate synthase in the leaves of intercropped wheat maintained a higher level than those of sole-cropped wheat (**Figures 1E, F**). In addition, the level of plasma membrane lipid peroxidation in wheat leaves under intercropping and sole cropping were evaluated by measuring POD and MDA. The POD activity was significantly (*p* < 0.05) increased in wheat leaves in both cropping patterns, but the levels in intercropped wheat were significantly higher than those in sole-cropped wheat (**Figure 1G**); the MDA content was significantly (*p* < 0.05) decreased in wheat leaves in both cropping patterns, but the levels in intercropped wheat were significantly (*p* < 0.05) lower than those in sole-cropped wheat (**Figure 1H**), implying that the sole cropping resulted in severe plasma membrane damage in wheat leaves. These results suggested that a high level of photosynthetic capacity, nutrient accumulation, and low level of lipid peroxidation might contribute to the high production of wheat under intercropping pattern.

**Figure 2:**
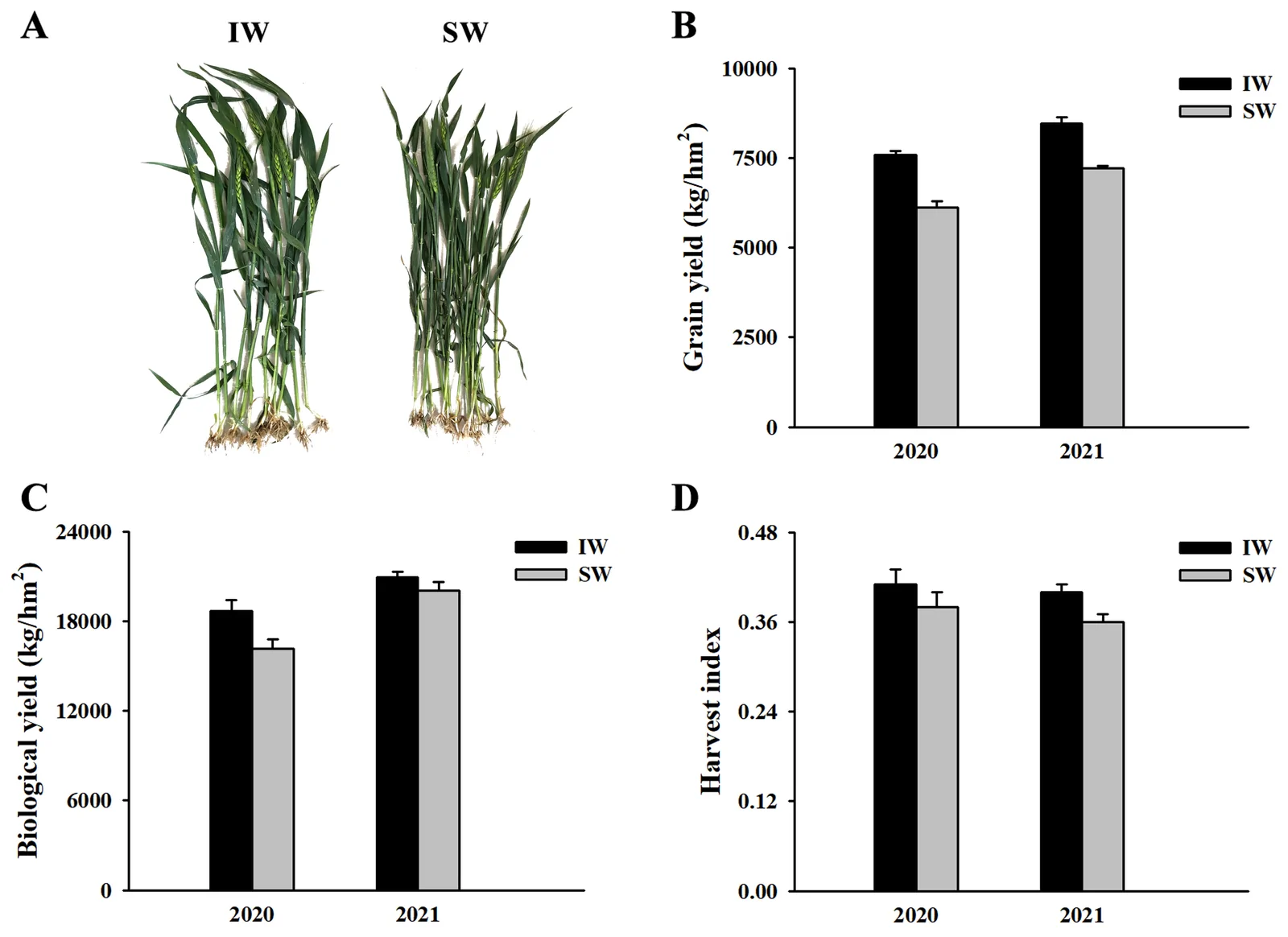
Growth characteristics and yield changes in wheat under different cropping patterns. **(A)**, Representative wheat plants grown under wheat–maize intercropping (IW) and sole cropping (SW). **(B)**, Grain yield of wheat under intercropping and sole cropping treatments. **(C)**, Biological yield of wheat under intercropping and sole cropping treatments. **(D)**, Harvest index of wheat under intercropping and sole cropping treatments. The values are means ± SD from three biological replicates (**p<0.01).

The intercropped wheat showed a plumper ear, and its ear leaves were significantly longer than those of sole-cropped wheat (**Figure 2A**). In the 2-year experiment, the intercropping significantly increased the grain yield and biological yield compared with the sole cropping (**Figures 2B, C**). In 2020, the grain yield and biological yield of intercropped wheat reached 7575.92 and 18683.89 kg·ha^-1^, while only 6127.1 and 16131.4 kg·ha^-1^ in sole-cropped wheat. In 2021, the grain yield and biological yield of intercropped wheat reached 8469.9 and 20948.1 kg·ha^-1^, while only 7204.5 and 20036.5 kg·ha^-1^ in sole-cropped wheat. In addition, similar results were obtained by the harvest index after intercropping treatment, which reached 0.41 and 0.40 in 2020 and 2021, respectively, while only 0.38 and 0.36 in sole-cropped wheat (**Figure 2D**).

Intercropping significantly increased the spike number per unit area by 27.4% in 2020 and 25.3% in 2021 compared with sole cropping (**Table 1**). In contrast, grain number per spike and thousand-grain weight did not differ significantly between cropping patterns in either year. These results suggest that the grain-yield advantage of intercropped wheat was associated primarily with a greater spike number per unit area rather than changes in grain number per spike or thousand-grain weight.

**Table 1:** Yield components of wheat under intercropping and sole cropping in 2020 and 2021.

Year	Cropping pattern	Spike number (spikes·m^-2^)	Grain number per spike	Thousand-grain weight (g)
2020	IW	514.92 ± 2.19 a	31.10 ± 0.51 a	48.60 ± 0.57 a
	SW	404.32 ± 3.53 b	31.78 ± 0.56 a	46.80 ± 1.96 a
2021	IW	536.91 ± 1.71 a	31.96 ± 1.43 a	47.25 ± 1.04 a
	SW	428.42 ± 2.58 b	31.52 ± 0.67 a	45.19 ± 1.78 a

Values are means ± SD of three independent field replicates. IW, wheat grown under wheat–maize intercropping; SW, sole-cropped wheat. Different lowercase letters indicate significant differences between treatments within the same year at *p* < 0.05.

### 3.2 Quality control analysis of wheat transcriptome

**Figure 3:**
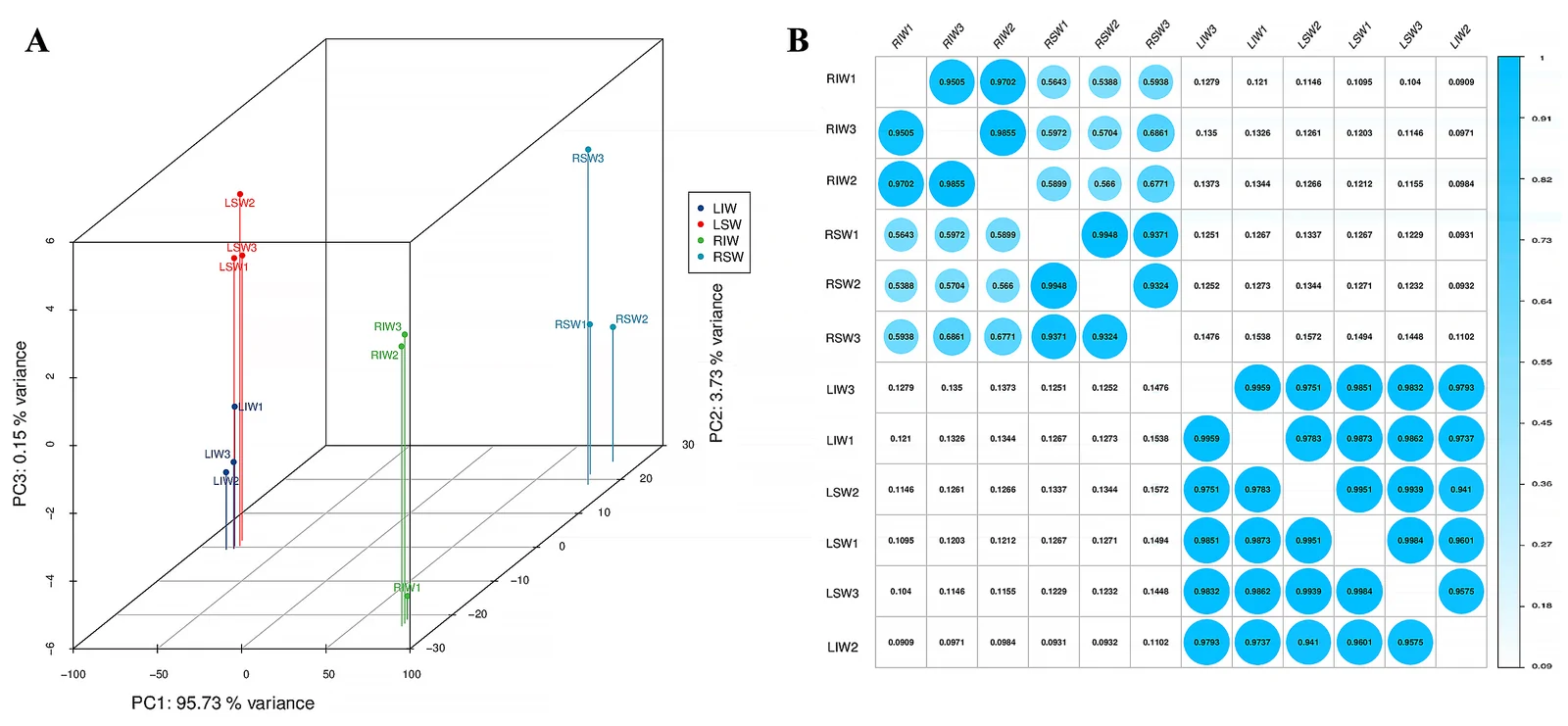
PCA and correlation coefficient map of wheat under different cropping patterns. **(A)**, PCA of detected genes in transcriptome; **(B)**, Correlation coefficient of detected samples in transcriptome.

At the wheat booting stage in 2021, 45.54–50.30 million clean reads were obtained from each library. The Q30 values ranged from 94.23% to 94.40%, while the total and unique mapping rates ranged from 81.89% to 96.82% and from 75.45% to 85.99%, respectively (**Supplementary Table 2**). A total of 84,140 genes were detected across the four sample groups (**Supplementary Table 3**). The PCA showed a good agreement among three biological replicates of LIW, LSW, RIW, and RSW (**Figure 3A**), and all replicates exhibited a high overlap in transcriptome for each sample (**Supplementary Figure S1**). In addition, a repeatability analysis between three biological replicates of LIW, LSW, RIW, and RSW samples indicated that their correlation coefficients were greater than 0.9 (**Figure 3B**). These results suggested a high level of reliability of the transcriptome analysis.

### 3.3 Quantitative transcriptomic analysis of wheat in different cropping patterns

**Figure 4:**
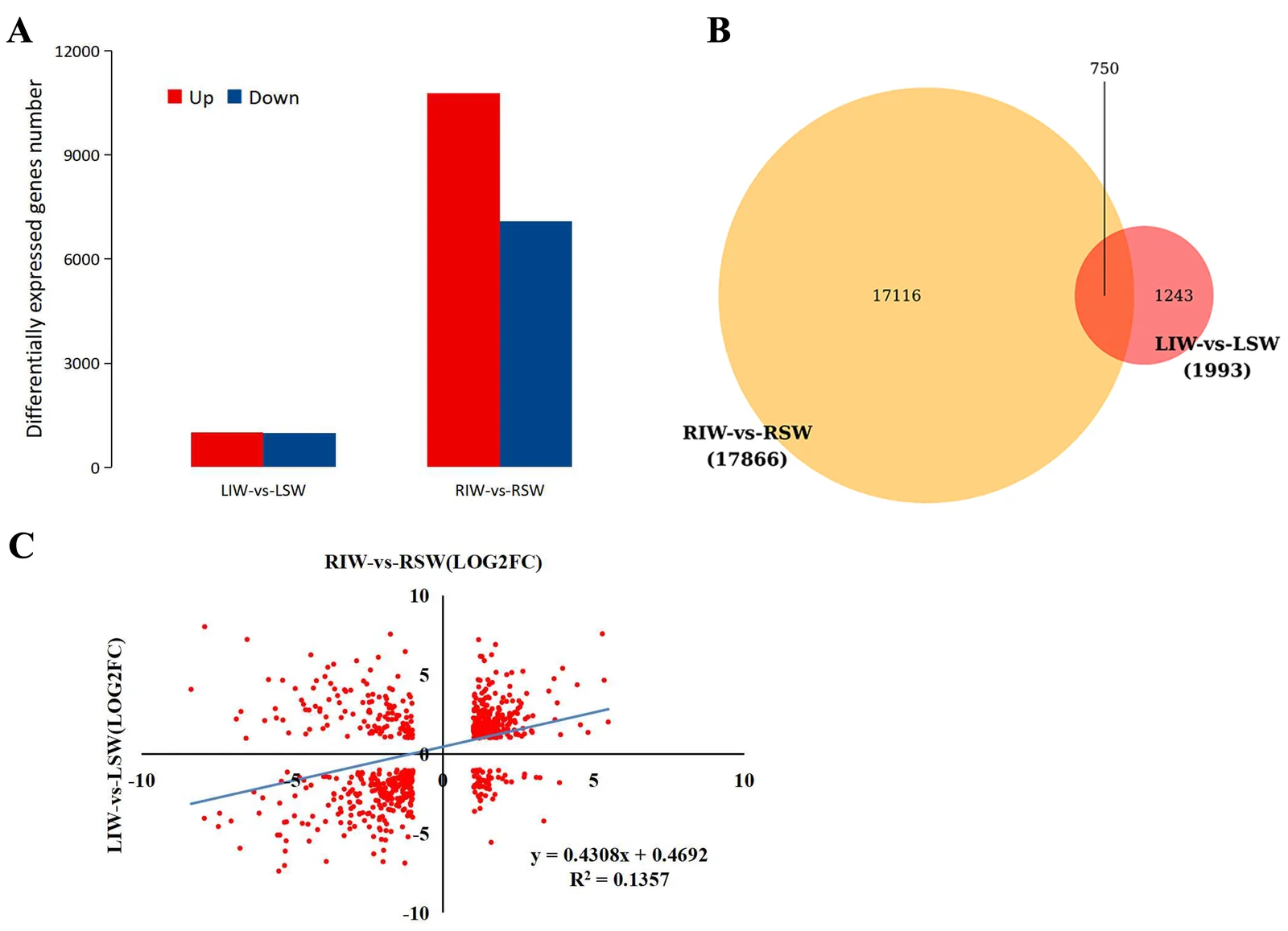
Comparison of transcripts in leaves and roots of wheat under intercropping treatment. **(A)**, The number of DEGs identified in leaves and roots of wheat; **(B)**, Venn diagram of identified DEGs in leaves and roots of wheat; **(C)**, Expression patterns of shared DEGs in leaves and roots of wheat.

To identify differentially expressed genes (DEGs) associated with intercropping at the booting stage in 2021, transcript levels in wheat leaves and roots were compared between the two cropping patterns. Under the intercropping pattern, a total of 1993 (up, 1005; down, 988) genes were found to be differentially expressed in wheat leaves, and 17866 (up, 10782; down, 7084) genes were differentially expressed in wheat roots in comparison with sole cropping (**Figure 4A**; **Supplementary Table 4**). Notably, the number of DEGs was significantly changed in the leaves and roots of wheat, suggesting great differences in gene expression in both tissues. Furthermore, 750 shared DEGs were found in leaves and roots of wheat; among these shared DEGs, 363 were up-regulated and 387 were down-regulated in leaves, while 430 were up-regulated and 320 were down-regulated in roots (**Figure 4B**; **Supplementary Table 5**). In addition, the expression patterns of shared DEGs in leaves and roots were analyzed using Pearson’s correlation test. There was a significant positive correlation between the fold-changes of shared DEGs, with a Pearson correlation coefficient of 0.368 (**Figure 4C**). These results indicated that the expression patterns of most genes were consistent, which might play a synergistic role in different tissues of intercropped wheat.

### 3.4 GO annotation and KEGG analysis of DEGs in intercropped wheat

**Figure 5:**
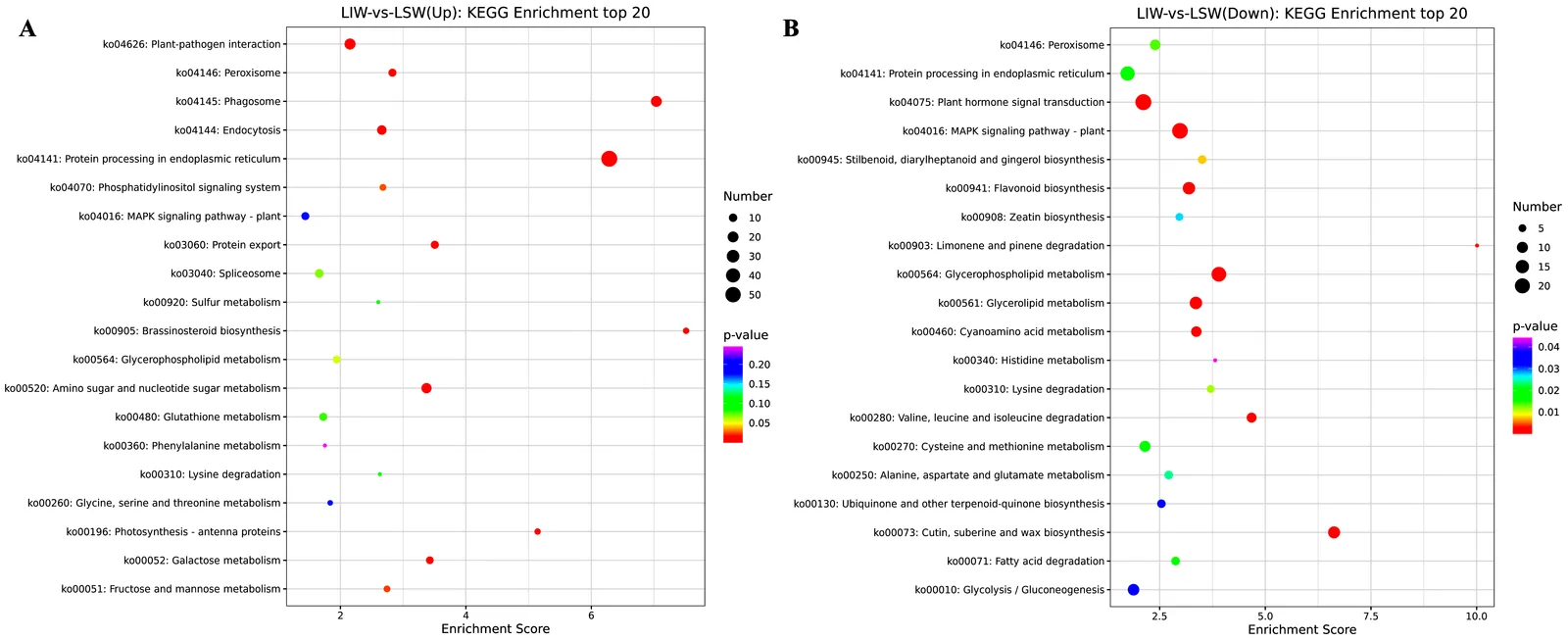
KEGG pathway enrichment analysis of DEGs identified in wheat leaves under intercropping. **(A)** Top 20 KEGG pathways enriched by upregulated DEGs in the LIW vs. LSW comparison. **(B)** Top 20 KEGG pathways enriched by downregulated DEGs in the LIW vs. LSW comparison.

**Figure 6:**
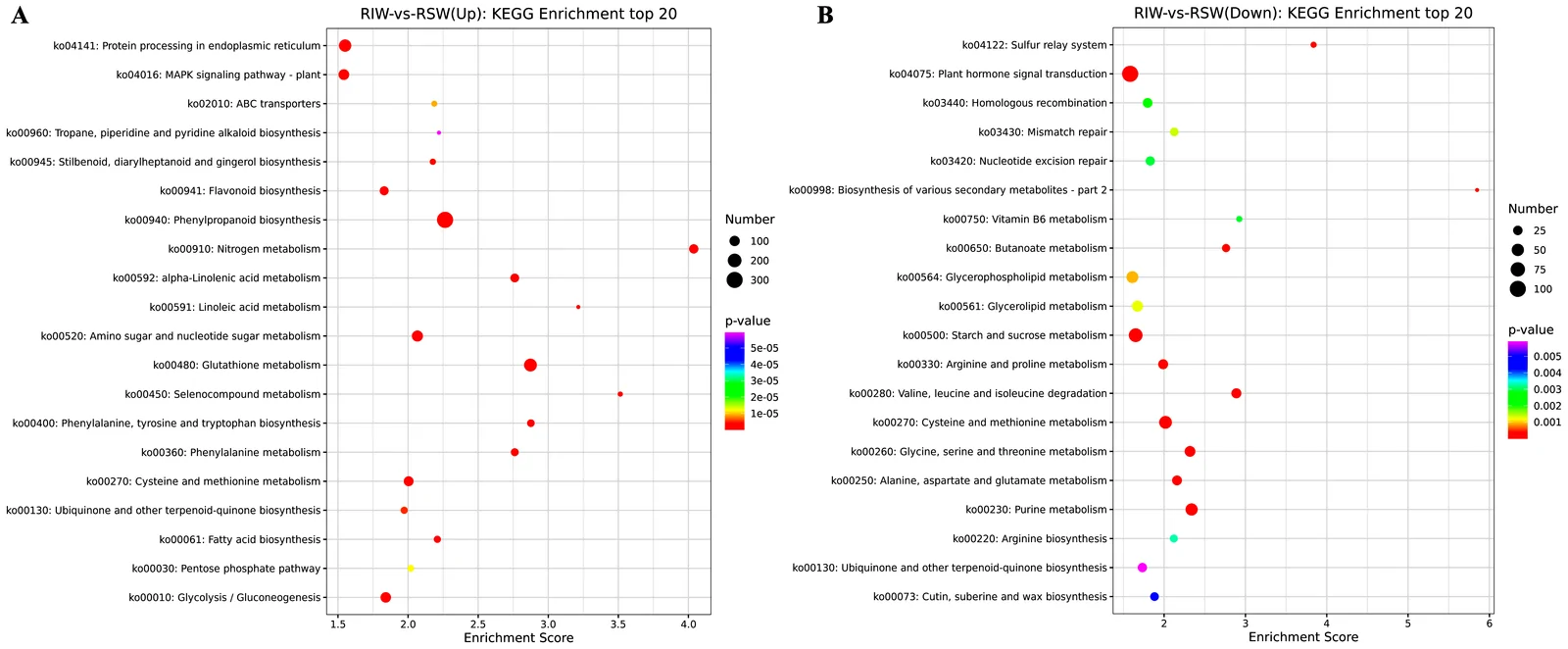
KEGG pathway enrichment analysis of DEGs identified in wheat roots under intercropping. **(A)** Top 20 KEGG pathways enriched by upregulated DEGs in the RIW vs. RSW comparison. **(B)** Top 20 KEGG pathways enriched by downregulated DEGs in the RIW vs. RSW comparison.

The DEGs identified in the leaves and roots of intercropped wheat were subjected to GO classification annotation and KEGG analysis (**Supplementary Figure S2**; **Supplementary Tables 6**, **S7**). Analysis of the top 20 enriched KEGG pathways showed that protein processing in endoplasmic reticulum (ko04141), plant-pathogen interaction (ko04626), brassinosteroid biosynthesis (ko00905), photosynthesis (ko00196), and peroxisome (ko04146) pathways were significantly (*p* < 0.005) enriched by up-regulated DEGs identified in leaves (**Figure 5A**), whereas cutin, suberine and wax biosynthesis (ko00073), glycerophospholipid metabolism (ko00564), MAPK signaling pathway (ko04016), flavonoid biosynthesis (ko00941), and plant hormone signal transduction (ko04075) were significantly (*p* < 0.001) enriched by down-regulated DEGs (**Figure 5B**). In roots, protein processing in endoplasmic reticulum (ko04141), phenylpropanoid biosynthesis (ko00940), glutathione metabolism (ko00480), alpha-linolenic acid metabolism (ko00592), flavonoid biosynthesis (ko00941), and fatty acid biosynthesis (ko00061) pathways were significantly (*p* < 0.001) enriched by up-regulated DEGs (**Figure 6A**), while plant hormone signal transduction (ko04075), starch and sucrose metabolism (ko00500), and cysteine and methionine metabolism (ko00270) were significantly (*p* < 0.001) enriched by down-regulated DEGs (**Figure 6B**). These pathways might be associated with the response of wheat to intercropping and therefore warrant further investigation.

### 3.5 RNA-seq validation by qRT-PCR

**Figure 7:**
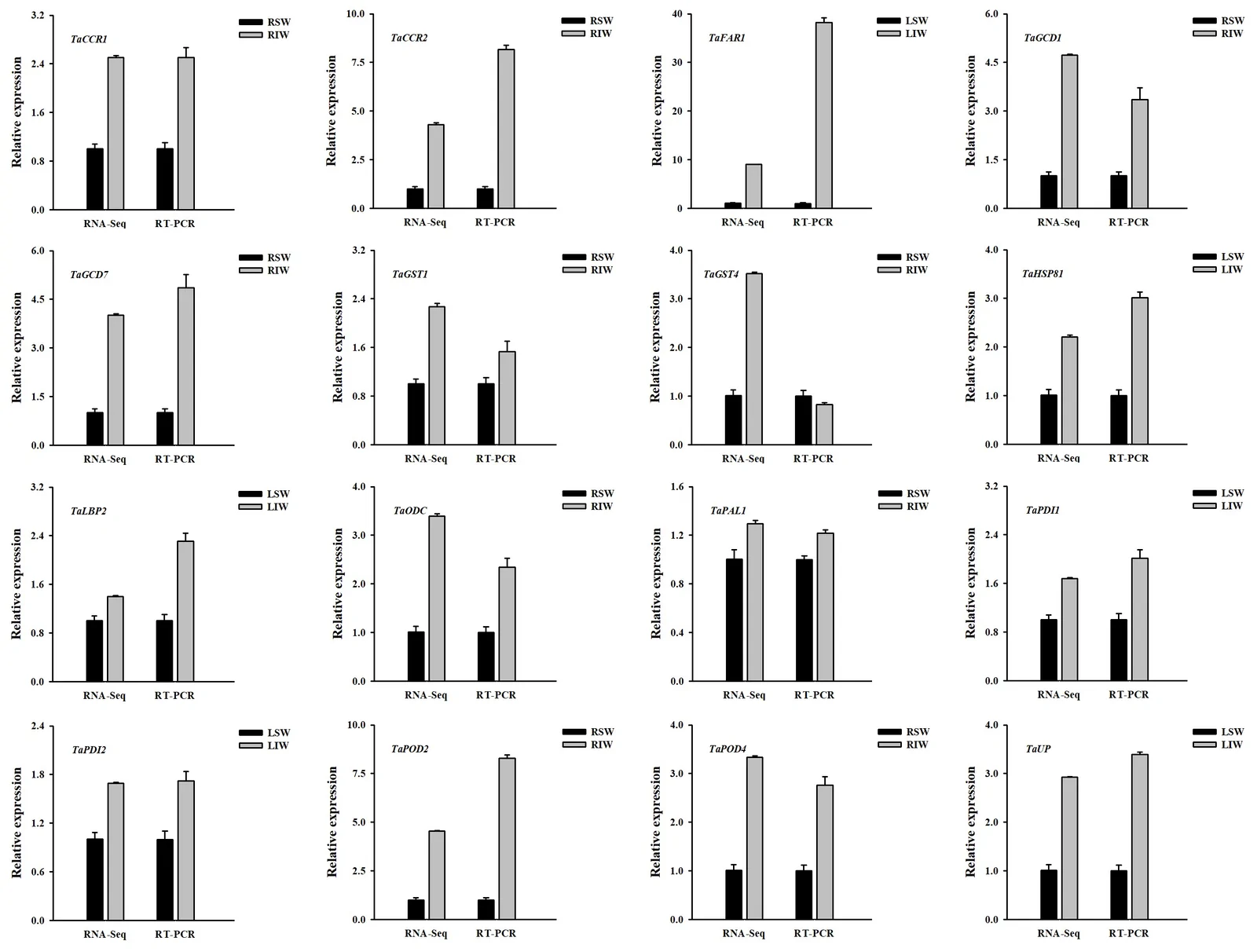
Comparative analysis of mRNA and RNA-seq levels in wheat under different cropping patterns. The values are means ± SD from three biological replicates in wheat under intercropping treatment.

Based on the high fold-change of DEGs, the qRT-PCR analysis was used to validate the expressions of *TaCCR1*, *TaCCR2*, *TaFAR1*, *TaGCD1*, *TaGCD7*, *TaGST1*, *TaGST4*, *TaHSP81*, *TaLBP2*, *TaODC*, *TaPAL1*, *TaPDI1*, *TaPDI2*, *TaPOD2*, *TaPOD4*, *UP* candidate intercropping-responsive genes identified in leaves and roots of wheat. 15 out of 16 DEGs were consistent at both the mRNA and RNA-seq levels in intercropped wheat compared to sole-cropped wheat. Beyond that, *GST4* genes showed discordance between the mRNA and RNA-seq levels (**Figure 7**). These results suggested that qRT-PCR expression patterns were in line with the RNA-seq, and the RNA-seq results were dependable in the present study. The discrepancy observed for *TaGST4* might be related to differences in the sensitivity and normalization procedures of the two methods.

## 4 Discussion

The maize and wheat intercropping system is becoming increasingly popular around the world for its high production and good harvest. Leaves are the crucial sites of photosynthesis, which contribute to the biosynthesis of plant biomass and energy (Makino, 2011). In the past decade, studies have focused on the effects of wheat-maize intercropping on the productivity formation of crops (Gou et al., 2016; Yin et al., 2020). However, these studies have mainly focused on agronomic, physiological, and ecological responses. Although transcriptomic analysis has been applied to characterize stress signaling, secondary metabolism, and carbon–nitrogen regulation in maize rhizospheres under intercropping (Zhang et al., 2024), the tissue-specific transcriptional responses of wheat leaves and roots remain insufficiently understood. In the present study, two-year field measurements were integrated with physiological, biochemical, and transcriptomic analyses of wheat leaves and roots to examine wheat responses to wheat–maize intercropping at multiple biological levels. This integrated approach might provide additional information on the biological processes associated with wheat performance under intercropping and contribute to a more comprehensive understanding of the wheat–maize intercropping system.

### 4.1 Comparison of effects of cropping patterns on wheat physiology and biochemistry

Intercropping is the behavior of growing two or more crops in the same field simultaneously, which have different heights and canopy structures (Willey, 1990). These differences between the crops benefit light harvesting to maximize crop production (Wang et al., 2017b). Intercropping can result in various changes in crop growth and physiology (Gong et al., 2020; Yin et al., 2021). Liang et al. (2016) showed that rice’s (*Oryza sativa* L.) chlorophyll content and the net photosynthetic rate increased in the rice/water spinach (*Ipomoea aquatica* F.) intercropping system. In addition, numerous studies have suggested that crop canopy structure was closely associated with photosynthetically active radiation (PAR) utilization (Feng et al., 2016; Charbonnier et al., 2017; Cheng et al., 2022). In this study, wheat’s photosynthetic rate and SPAD value in the intercropping system were significantly higher than in sole cropping (**Figures 1A–D**). These advantages for intercropping might be due to a combination of increased light harvest and better canopy structure. Belowground complementarity may also contribute, as vertical root stratification can reduce water competition between wheat and maize and improve wheat yield under intercropping (Shi et al., 2025). These results are in accordance with the findings in cucumber (*Cucumis sativus* L.)/wheat and maize/pea (*Pisum sativum* L.) intercropping systems (Liu et al., 2019; Yang et al., 2022). It has been reported that sucrose phosphate synthase, a vital enzyme responsible for sucrose synthesis, promotes plant growth and biomass accumulation (Maloney et al., 2015). This study found a higher level of SPS activity and protein content in intercropped wheat compared to sole-cropped wheat (**Figures 1E, F**), which implied that intercropping might increase the biosynthesis of sucrose and protein in leaves of wheat, leading to more biomass accumulation. Under hostile environmental conditions, the antioxidant enzymes are essential for maintaining normal cellular redox homeostasis (Ray et al., 2012). In the present study, POD activity showed an increasing trend in three developing stages under both the cropping patterns, and the levels of POD in intercropping were higher than those in sole cropping. In complete contrast to the POD activity, the MDA content exhibited a gradually decreasing trend, and the levels of MDA in the three developing stages of intercropping were lower than those in sole cropping (**Figures 1G, H**), which was consistent with the observation that the strip intercropped eggplant (*Solanum melongena* L.) with garlic (*Allium sativum* L.) showed a lower level of MDA compared with sole cropping (Wang et al., 2015). The enhanced POD activity and weakened lipid peroxidation implied that the wheat in intercropping treatment could maintain a normal redox environment for leaf growth and development, thus suffering less damage from the environment.

### 4.2 Comparison of effects of cropping patterns on growth characteristics and production of wheat

Intercropping contributes to the success of traditional and modern agriculture, providing a natural barrier to disease and increasing biodiversity (Lopes et al., 2016; Zhu and Morel, 2019). In general, component crops in an intercropping system differ in productivity. Many studies have reported yield advantages or disadvantages in different intercropping component crops compared with sole cropping (Raseduzzaman and Jensen, 2017; Yin et al., 2017; Xiao et al., 2018). For example, rice/water spinach intercropping improved rice production but reduced water spinach yield (Ning et al., 2017). The maize and soybean intercropping contributed to the accumulation of maize yield compared with sole cropping (Liu et al., 2017). Recent evidence further indicates that the yield response of wheat in wheat–maize intercropping depends on interspecific interactions and management practices, including the relative sowing time of the component crops (Sun et al., 2024).

This study found that the size of ears in intercropped wheat was expanded, and that the length of ear leaves was lengthened by intercropping treatment (**Figure 2A**). Furthermore, intercropping raised wheat grain yield by 19.12% and 14.94% in 2020 and 2021, respectively (**Figures 2B, C**). Consistently, wheat–maize intercropping increased the wheat harvest index compared with sole cropping (**Figure 2D**). Grain yield is jointly determined by spike number per unit area, grain number per spike, and thousand-grain weight. In the present study, the yield advantage of intercropped wheat was accompanied by increases of 27.4% and 25.3% in spike number per unit area in 2020 and 2021, respectively, whereas grain number per spike and thousand-grain weight were not significantly affected (**Table 1**). These results suggest that the higher grain yield of intercropped wheat was associated primarily with the formation or retention of a greater number of productive spikes per unit area rather than substantial changes in grain number per spike or individual grain weight. The increased spike density may be related to improved tiller establishment and survival under the favorable resource conditions created by intercropping, although this possibility requires further investigation. This interpretation is consistent with recent studies showing that effective spike number is an important determinant of grain yield in irrigated spring wheat and wheat-based intercropping systems, although the relative contribution of individual yield components may vary with cropping pattern, environmental conditions, and field management (Li et al., 2025; Wang et al., 2025; Pampana et al., 2026). Collectively, these findings indicate that wheat–maize intercropping may provide a more favorable and stable growing environment, thereby promoting wheat growth and yield formation.

### 4.3 Comparison of effects of cropping patterns on wheat transcriptomic

Photosynthesis is a major driver of plant growth and biomass production (Nowicka et al., 2018). Brassinosteroids (BRs), a group of plant steroid hormones, are crucial for plant growth and development (Fujiyama et al., 2019). A large number of studies have shown that BRs can enhance photosynthesis and the activities of antioxidant enzymes (Zhou et al., 2014; Xia et al., 2018). In this study, transcriptomic analysis showed that photosynthesis-related genes were significantly enriched among the upregulated DEGs in intercropped wheat leaves (**Supplementary Table 7**), which was consistent with the higher photosynthetic rate observed under intercropping (**Figure 1**). In addition, an upregulated DEG encoding a cytochrome P450 involved in brassinosteroid biosynthesis was identified. These are consistent with what has been found in previous research, which suggested that intercropped could improve photosynthetic capacity in cucumber/wheat intercropping system (Liu et al., 2019). In addition, Zhan et al. (2021) reported that GW10, a member of P450 subfamily, regulated grain size and number in rice. In the present study, one key enzyme of BR biosynthesis, cytochrome P450, was found and up-regulated in the leaves of intercropped wheat (**Supplementary Table 7**). Therefore, we speculate that BR-related transcriptional responses might be associated with photosynthetic processes and biomass accumulation under intercropping, which may contribute to the higher biological yield observed under intercropping (**Figure 2**). However, brassinosteroid concentrations and signaling activity were not directly measured and require further validation.

It has been reported that calreticulin (CRT) is required for calcium homeostasis, which is extensively expressed in all eukaryotic organisms and has a highlighted significance in plant growth and development as well as biotic and abiotic stress responses (Jia et al., 2009; Suwińska et al., 2017). Li et al. (2021) reported that the heat-shock protein 70 (HSP70) was correlated positively with auxin-mediated embryo development in Arabidopsis (*Arabidopsis thaliana* L.). HSP90, as a molecular chaperone, is considered indispensable for the maintenance of cellular homeostasis under natural growth conditions; their levels rise as their transcription and translation are enhanced (Tichá et al., 2020). Our results showed that genes encoding CRTs and HSPs were up-regulated in leaves of intercropped wheat and accompanied by a high level of soluble protein content (**Supplementary Table 7**; **Figure 1F**). In addition to CRTs and HSPs, several up-regulated DEGs encoding luminal-binding proteins and protein disulfide isomerases were identified in the leaves and roots of intercropped wheat. These transcriptional changes suggest that protein synthesis and processing may respond to intercropping, although the corresponding changes at the protein level remain to be verified.

Plant secondary metabolites are essential for adapting to environmental stresses and defense against pathogens (Ramakrishna and Ravishankar, 2011; Chen et al., 2021). Phenylpropanoid compounds assemble a large class of secondary metabolites that originate from phenylalanine and are beneficial to defense against various plant stresses (Liu et al., 2015). Similar pathway-level responses have been reported in other cereal intercropping systems, including changes in stress signaling and secondary metabolism in maize rhizospheres (Zhang et al., 2024) and defense-, flavonoid-, and phenylpropanoid-related processes in rice roots (Han et al., 2025). These findings support the possible involvement of similar processes in wheat responses to intercropping, although further functional validation is required. In the present study, some up-regulated DEGs in intercropped leaves, such as encoding disease resistance protein, calmodulin-like protein, heat shock protein 81/90, and respiratory burst oxidase homolog protein were significantly enriched in plant-pathogen interaction pathway (**Figure 5A**; **Supplementary Table 7**), implying that its defense system was strengthened under intercropping treatment. A further novel finding was that the phenylpropanoid biosynthesis pathway was significantly enriched by some up-regulated DEGs in intercropped roots, encoding phenylalanine ammonia-lyase, beta-glucosidase, caffeoyl-CoA O-methyltransferase, peroxidase, and 4-coumarate-CoA ligase (**Figure 6A**; **Supplementary Table 7**), suggesting that the production of secondary metabolites was accumulated under intercropping treatment compared to the sole cropping. It is in agreement with those reported by Duan et al (Duan et al., 2021). These results indicated that accumulated secondary metabolites and enhanced plant-pathogen interaction together contributed to the formation of grain yield and biological yield of intercropped wheat.

Peroxisomes are involved in multiple metabolic processes, including fatty acid oxidation, ether lipid synthesis, and reactive oxygen species (ROS) metabolism (He et al., 2021). Under adverse environmental conditions, ROS-scavenging enzymes are necessary to maintain normal cellular oxidative homeostasis (Ray et al., 2012). Glutathione is well known for its ROS scavenging function in biotic stress (Cheng et al., 2015). In this study, some DEGs belonging to the peroxisome pathway were significantly enriched in intercropped leaves (**Figure 5A**; **Supplementary Table 7**). Another promising finding was that the glutathione metabolism pathway was significantly enriched by DEGs, and all glutathione S-transferases (GST) enriched in this pathway were up-regulated in intercropped roots (**Figure 6A**; **Supplementary Table 7**). Additionally, three peroxidases with accumulated expression in intercropped roots compared to the sole cropping were consistent with the determination of POD enzyme activity (**Figure 1G**; **Figure 7**). Altogether, these results suggest that peroxisome and glutathione-related transcriptional responses may be associated with redox regulation in intercropped wheat. However, the corresponding changes in glutathione content and antioxidant protein abundance were not directly measured and require further verification.

It was well known that biotic and abiotic stresses could result in the release of alpha-linolenic acid, an essential precursor for the biosynthesis of jasmonic acid (Yan et al., 2013), which interacted with other plant hormones to regulate plant growth, secondary metabolism, pathogen infection, and tolerance to abiotic stresses (Hu et al., 2017b). In this study, all DEGs implicated in the alpha-linolenic acid metabolism were up-regulated in intercropped roots, including lipoxygenases, allene oxide cyclase, allene oxide synthase, alcohol dehydrogenase, 12-oxophytodienoate reductase, and 12-oxophytodienoic acid reductase. These transcriptional changes suggest that alpha-linolenic-acid- and jasmonate-related processes may participate in the root response to intercropping. However, jasmonate concentrations were not measured, and this interpretation requires further validation.

It should be noted that the transcriptomic analysis was conducted only on wheat leaves and roots collected at the booting stage in 2021. Transcriptomic analyses across additional developmental stages and growing seasons will be required to determine the temporal stability of these responses.

## 5 Conclusion

In summary, we characterized the transcriptional profiles of wheat leaves and roots at the booting stage in 2021 and integrated these results with physiological and agronomic observations obtained during two growing seasons. The significant enrichment of pathways related to photosynthesis, protein processing in the endoplasmic reticulum, phenylpropanoid biosynthesis, glutathione metabolism, and alpha-linolenic acid metabolism under intercropping might play important roles in promoting wheat growth and yield formation. These findings provide candidate biological processes for further functional validation and may contribute to a better understanding of wheat responses to wheat–maize intercropping.

## Data Availability

The raw data in this study has been deposited in the National Center for Biotechnology Information (NCBI), repository link: https://www.ncbi.nlm.nih.gov. The BioSample/SRA accession for the representative sample is SAMN38242314/SRS19549730.

## Supplementary material

Supplementary Figure 1 The reproducibility analysis between three biological replicates of LIW, LSW, RIW, and RSW samples in transcriptome.

Supplementary Figure 2 GO classification analysis of DEGs identified in leaves **(A)** and roots **(B)** of wheat.

Supplementary Table 1 The primer sequences for qRT-PCR.

Supplementary Table 2 The mapping rate of transcriptome in reference genome.

Supplementary Table 3 The list of all the identified genes in transcriptome.

Supplementary Table 4 The list of DEGs identified in leaves and roots of wheat.

Supplementary Table 5 DEGs jointly owned in leaves and roots of wheat.

Supplementary Table 6 GO classification annotation by the DEGs in leaves and roots of wheat.

Supplementary Table 7 KEGG pathways enriched by the DEGs in leaves and roots of wheat.
