# Supplementary material for: Comprehensive analysis of physiological characteristic and global transcriptional profiling to unravel the mechanism of wheat yield increase in intercropping
Source: Front Plant Sci. 2026 Aug 10;17:1857726. doi: 10.3389/fpls.2026.1857726 (PMC13500323; doi:10.3389/fpls.2026.1857726)
Supplement: Supplementary file 1 [file DataSheet2.docx]

Supplementary Material

## 1.1 Supplementary Figures

**Supplementary Figure 1.** The reproducibility analysis between three biological replicates of LIW, LSW, RIW, and RSW samples in transcriptome.

**Supplementary Figure 2.** GO classification analysis of DEGs identified in leaves (A) and roots (B) of wheat.
